# Co-producing an intervention to prevent mental health problems in children and young people in contact with child welfare services

**DOI:** 10.1186/s12889-024-19770-6

**Published:** 2024-08-21

**Authors:** Ruth McGovern, Abisola Balogun-Katung, Benjamin Artis, Hayley Alderson, Eric Brown, Tim Diggle, Raghu Lingam, Paul McArdle, Judith Rankin, Paige Thomason, Eileen Kaner

**Affiliations:** 1 Population Health Sciences Institute, Newcastle University, Baddiley-Clark Building, Richardson Road, Newcastle upon Tyne, Newcastle upon Tyne, NE2 4AX UK; 2 Children and Young People’s Primary Care Mental Health Service, Tyne and Wear NHS Foundation Trus, Cumbria, Northumberland UK; 3 Children’s Social Care, Newcastle upon Tyne, Newcastle City Council, UK; 4 Population Child Health Research Group, School of Women and Children’s Health, University, NSW, Australia; 5 Child and Adolescent Mental Health Services, Tyne and Wear NHS Foundation Trust, Newcastle upon Tyne, Cumbria, Northumberland UK; 6 Children’s Social Care, Cumberland Council, Northumberland UK

**Keywords:** Children and young people, Mental health, Adversity, Trauma-informed, Secondary prevention, Intervention development

## Abstract

**Background:**

Children and young people (CYP) in contact with child welfare services are at high risk of developing mental health problems. There is a paucity of evidenced-based preventative interventions provided to this population.

**Objective:**

This project worked in partnership with CYP, their parents/caregivers and the professionals who support them to co-produce a preventative mental health intervention for CYP in contact with child welfare services.

**Participants and setting:**

We recruited a purposive sample of CYP in contact with child welfare services (*n* = 23), parents/caregivers (*n* = 18) and practitioners working within child welfare services and mental health services (*n* = 25) from the North East of England and convened co-production workshops (*n* = 4).

**Methods:**

This project followed the established principles for intervention development, applying the six steps to quality intervention development (6SQUID) approach. The mixed method research consisted of four work packages with continuous engagement of stakeholders throughout the project. These were: a systematic review of reviews; focus groups with practitioners; interviews with parents/caregivers and CYP; co-production workshops.

**Results:**

We identified that the primary risk factor affecting CYP in contact with child welfare services is the experience of childhood adversity. The quality of relationships that the CYP experiences with both their parent/caregivers and the professionals involved in their care are considered to be the main factors amenable to change.

**Conclusions:**

We found that a trauma-informed, activity-based intervention with an embedded family-focused component provided to CYP who have experienced adversity is most likely to prevent mental health problems in those in contact with child welfare services.

**Supplementary Information:**

The online version contains supplementary material available at 10.1186/s12889-024-19770-6.

## Background

There is growing concern about the prevalence of mental health problems in children and young people (CYP) worldwide. In the UK, a national survey found that 1 in 9 children aged 5–16 years had a probable mental health problem in 2017 [1]. This rate then rose to 1 in 6 in 2020 [2], and has remained stable in 2021 and 2022 [3]. The prevalence of mental health problems, however, is not evenly distributed within society. CYP people in contact with child welfare services are particularly vulnerable to experiencing mental health problems. A recent meta-analysis estimated that 49% of children and adolescents in out-of- home placements have a mental health disorder [4] and child mental health is the third most common risk factor identified in all ‘Child in Need’ assessments conducted in the UK [5]. These CYP experience poor outcomes including an increased risk of substance use [6], involvement in offending behaviour [7], difficulties in their relationships with family and friends [8], diminished educational opportunities, [9] and unplanned pregnancy/parenthood [10]. Further, many go on to experience persistent mental health problems into adulthood [11] and reduced life opportunities [12], creating a pathway for disadvantage [13].

Child and Adolescent Mental Health Services (CAMHs) provide treatment to CYP experiencing moderate to severe mental health problems in the UK. However, it has been shown that treatment services are often not the most appropriate services for CYP in contact with child welfare services [14], who maybe ambivalent about help-seeking [15]. Mental health treatment services may not take account of the structural and material disadvantage experienced by the family [16]. There is a high prevalence of conduct difficulties in this population, which can be challenging for health focused systems to respond to [17]. CYP in contact with child welfare services often find the retelling of their stories to be difficult and potentially retraumatising [18]; they typically experience substantial mistrust [19] and prefer to invest in one relationship with a lead professional [14]. Further, long waiting lists for mental health services have been reported to be a deterrent to seeking help in the first instance, as well as negatively impacting upon engagement with mental health services once offered [14, 20]. A consequence of these barriers is that many CYP who are in contact with child welfare services do not have their mental health needs met [21].

To better respond to the mental health needs of CYP in contact with child welfare services, interventions should be tailored for this population as part of a holistic response around the person [22]. This response should further take account of their social context [23]. CYP come into contact with child welfare services for a wide variety of reasons including special education needs and disability, physical disability, unaccompanied children seeking asylum, family dysfunction, family break down and child maltreatment [5]. Each of these contexts introduces different risk factors which increase the likelihood of CYP experiencing mental health problems and the type of mental health care they need. Secondary prevention interventions have a large evidence base which report improvements in child mental health outcomes [24]. These interventions are typically delivered by generalist practitioners outside of specialist mental health services as part of a three-tier prevention framework. Whilst primary prevention is an intervention provided to whole populations prior to the development of a disease or health condition, and tertiary prevention is an intervention provided when a disease is established, secondary prevention is a targeted intervention provided in situations of elevated risk but before clinical thresholds for mental health disorders are met [25]. However, there is a paucity of research examining secondary mental health prevention interventions for CYP in contact with child welfare services. Preventative interventions within this context may offer an opportunity to intervene within situations of elevated mental health risk, address poor access to mental health services [26] and prevent the development of persistent mental health problems in this population [27]. However, little is known about the best approach to providing a secondary preventative intervention to CYP in contact with child welfare services including which CYP to focus support upon, the risk and protective factors that may be malleable to change and how to achieve change in these factors. This paper reports on a project which aimed to co-produce a secondary preventative intervention for CYP in contact with child welfare services, who are at risk of developing a mental health problem.

## Methods

**Fig. 1:**
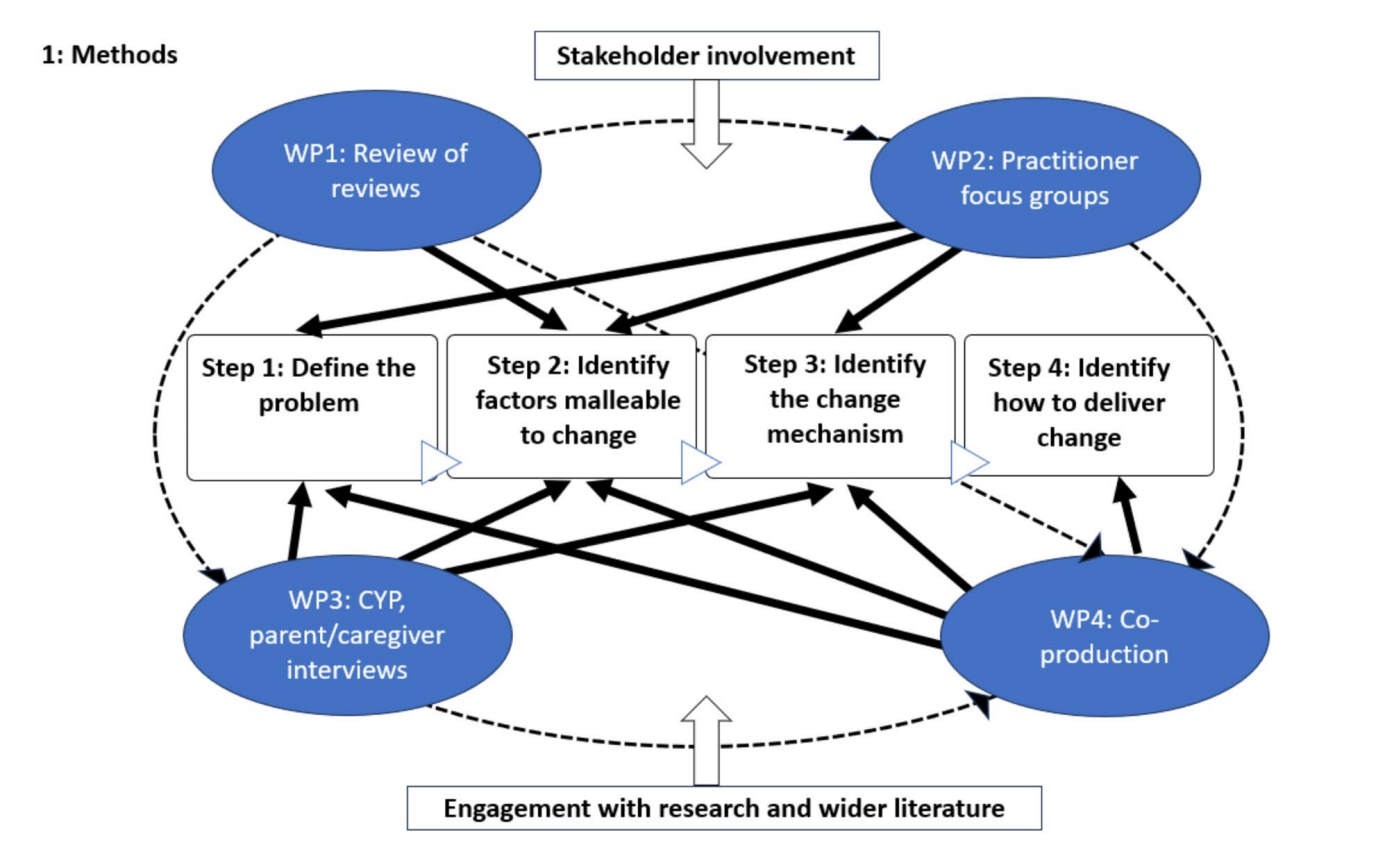
Methods

This project utilised a critical realist epistemological stance [28, 29], recognising the context-dependent nature of understanding. We followed established guidance for intervention development [30, 31] and adhered to reporting standards for intervention development studies [32]. We implemented the ten key actions recommended within intervention development [30]. These are: planning the development process; involving stakeholders; bringing together a team and establishing decision-making processes; reviewing published research evidence; drawing upon existing theories; articulating the programme theory; undertaking primary data collection; understanding context; paying attention to future implementation of the intervention in the real world; designing and refining the intervention. For simplicity, we present our methods and overall process as a series of actions. However, intervention development is not a linear process [30] and, as such, these actions were addressed in a dynamic, iterative way throughout the project, as illustrated in Fig. 1.

As recommended within the key action of planning of the development process, we drew upon published approaches to intervention development [30], applying the ‘six steps in quality intervention development’ (6SQUID) method [33] and embedding this within on-going stakeholder involvement [31] and co-production [34]. Based upon the taxonomy of intervention development approaches, we define our method as a combination of a stepped approach and a partnership approach [35]. CYP in contact with child welfare services are a highly heterogeneous group, with the ‘problem’ of mental health in this population being similarly multifaceted and complex. As such, the stepped approach detailed within the 6SQUID provided an opportunity to understand the mental health need within its context [36] as recommended by the updated Medical Research Council guidance [31], whilst becoming progressively focused upon specific subgroups within the population, risks and factors that are deemed malleable to change [33, 36]. Our continuous stakeholder involvement and approach to co-production further enabled us to move back and forth between the steps to iteratively refine our understanding throughout the development process. To date, we have not piloted the intervention or gathered evidence of effectiveness, which are phases in complex intervention research which can lead to intervention refinement [31]. As such, this paper will report on the first four steps of the 6SQUID approach.

Our mixed method approach consisted of four work packages (WP):

- WP1: systematic review of reviews.
- WP2: focus groups with practitioners.
- WP3: semi-structured interviews with CYP and parents/caregivers.
- WP4: co-production workshops.

A favourable ethical opinion was granted by the Health Research Authority West Midlands – Coventry & Warwickshire Research Ethics Committee (reference 22/WM/0034) on 28th March 2022. All research participants provided informed consent to participate (aged 16 years and over). Children and young people under the age of 16 years provided assent to participate and informed consent was provided by their parent/legal guardian.

### Work package 1: systematic review of reviews

We conducted a systematic review of systematic reviews to map available evidence relating to secondary preventative interventions and identify effective interventions to prevent mental health problems in CYP aged 3–17 years [24]. The review, which was guided by a pre-registered protocol (PROSPERO CRD42021290457), included systematic reviews of randomised controlled trials, quasi-experimental designs, and outcome evaluations of secondary preventative interventions (either selective or indicated) for children and young people aged 3–17 years or their parents/caregivers. We identified 49 unique systematic reviews (reported in 54 papers) which met our inclusion criteria. Each of the reviews included between 2 and 249 (mean 34) unique studies; the majority of which were reviews of only or mostly randomised controlled trials (70%). The reviews examined selective interventions (defined as interventions which are delivered to sub-group populations of young people at increased risk of mental health problems on the basis of biological, psychological, or social risk factors) (*n* = 22), indicated interventions (defined as interventions which target young people who are found to have pre-clinical symptoms) (*n* = 15) or a synthesis of both (*n* = 12). The certainty of the evidence in the reviews was rated as high, (*n* = 12) moderate (*n* = 5), low (*n* = 9) and critically low (*n* = 23), using the Assessment of Multiple Systematic Reviews (AMSTAR 2) tool [37].

### Work package 2: focus groups with practitioners

We conducted focus groups with child welfare and mental health practitioners across three local authority areas in the North East of England between April and May 2022 [38]. All consenting practitioners working with CYP from these service setting were considered eligible to participate. We initially planned to convene three focus groups to allow for data sufficiency across sites and capture variation by local authority areas. However, we identified a gap in our data around the impact of societal risk factors upon CYP and we convened a fourth focus group to examine this risk factor further. The final sample included a total of 25 practitioners (ranged from 4 to 9 practitioners per group). Practitioners were purposively sampled to achieve a maximum variation sample by service setting; mental health, children welfare service including early help, community safeguarding teams and those working with CYP in care/out-of-home placements. Practitioner characteristics are detailed in Table 1. A semi-structured topic guide was developed for the project to explore the risk and protective factors for mental health problems in children in contact with social care, their impact and consider factors that are malleable to change (the topic guide has been uploaded as a supplementary file).

### Work package 3: interviews with CYP and parents/caregivers

We conducted interviews with 23 CYP recruited via child welfare services and ‘Children in Care Councils’ (forums for children in out-of-home placement) and 18 interviews with parents/caregivers of CYP in contact with child welfare services in the North East of England between September 2022 and February 2023. CYP were eligible to participate if they were aged 11–25 years; were in contact with child welfare services and had experienced mental health concerns (self-reported). Parents/caregivers were eligible to participate if they provided care to a CYP who met the eligibility criteria. On-going recruitment decisions were informed by regular review of progress in achieving data saturation (see 2.4 data analysis for further details). The mean age of CYP who participated was 15 (range: 11-21yrs) and included both female (n = 12) and male (n = 11) participants. Most were White British (n = 19). The parents/caregivers were mostly mothers (n = 7) or residential care staff (n = 7). We also interviewed grandparents (n = 2) and foster carers (n = 2). The participant characteristics for CYP and parent/caregivers are detailed in Table 1. Analysed data from WP2 was used to inform the development of case vignettes utilised in interviews with CYP within WP3. All CYP selected one or more case vignettes (from a selection of four), which detailed hypothetical scenarios relating to the main risk and protective factors identified within practitioner focus groups within work package 2. The vignettes were implemented to promote the increasing focus upon the ‘problem’ whilst also promoting ethical data collection. Vignettes enabled CYP to discuss mental health risk, resilience and support in detail whilst not being required to disclose personal details which may be distressing to them. The participating CYP engaged in a semi-structured interview relating to the vignette, examining the mental health impact of this scenario, factors that they considered malleable to change and possible intervention approaches. In addition, 21 CYP completed an optional semi-structured exercise designed to support CYP to explore their own experiences of mental health problems. Parent/caregiver interviews all examined these topics as they related to their own experiences of caregiving to a CYP in contact with child welfare services who has mental health concerns. All case vignettes, topic guides and exercises have been uploaded used within this work package have been uploaded as a supplementary file.

**Table 1:** Participant characteristics

Practitioner characteristic	*N* = 25	%
Gender		
*Female*	24	96
*Male*	1	4
**Role**		
*Social worker*	11	44
*Family support worker*	5	20
*Clinical psychologist*	3	12
*Child support worker*	3	12
*Mental health (other)*	3	12
**Service setting**		
Children’s social care – community	12	48
Children in care	4	16
Youth justice	1	4
Early help	5	20
Mental health	3	12
**Child/young person characteristic**	*N* = 23	%
**Gender**		
*Female*	12	52
*Male*	**9**	**39**
*Non-binary*	**2**	**9**
**Age**		
*11–13 yrs*	10	44
*14–17 yrs*	7	30
*18–21 yrs*	6	26
**Ethnicity**		
*White British*	19	83
*Chinese*	1	4
*Black African*	1	4
*Black British*	1	4
*Mixed British*	1	4
**Setting**		
*Early help*	1	4
*Child in need*	10	44
*Child in care*	8	35
*Care leaver*	4	17
**Parent characteristic**	*N* = 18	**%**
**Gender**		
*Female*	15	83
*Male*	3	17
**Ethnicity** *White British*	18	100
**Family relation**		
*Mother*	7	39
*Grandparent*	2	11
*Foster parent*	2	11
*Residential carer*	7	39
**Setting**		
*Early help*	3	17
*Child in need*	3	33
*Child protection*	1	6
*Child in care*	8	44

### Data analysis: work package 3 and 4

The audio recorded focus group discussions and interviews were transcribed verbatim and uploaded to NVivo for data management. Data was analysed thematically [39] and guided by the socio-ecological model [40, 41]; a theoretical framework used to examine the complex interplay between individual, interpersonal, community and society level determinants of health. A thematic map depicting the data analysis for both work package 2 and 3 is available as a supplementary file.

Rigor within qualitative research is most often considered in terms of the trustworthiness, typically defined by the credibility, transferability, dependability, and confirmability of the processes (Guba & Lincoln, 1989). Two researchers (ABK and BA) coded the data in an iterative process where the team identified, discussed, and refined themes, which then informed subsequent analysis. We considered the credibility of the study in regular analysis meetings wherein we reflected upon the extent to which the interview process was capturing the social reality of the participants, and how far our findings would transfer to different contexts (such as different CYP age, gender, family circumstances, geographical locations). We finalised data collection once data saturation had been reached. Within the current study, data saturation is perceived as ‘a-priori thematic saturation’ [42]. Specifically, we iteratively applied a framework to our data that was informed by our stakeholder engagement and emerging findings from the study. We exemplified the findings at the level of lower-order codes or themes. We recognise that there is always potential for ‘new’ codes and themes to emerge [43] and therefore we did not consider data saturation to be a specific event. However, we acknowledged we had reached a stage of analysis where further data collection offered reduced potential to enhance understanding. We considered data saturation to have been achieved after three successive interviews did not result in data that added new and additional comprehension.

### Work package 4: co-production workshops

We presented our findings from WP1-3 at a regional children’s mental health conference held in June 2023, attended by practitioners, operational and strategic managers and leaders from both child welfare and mental health services. Delegates were invited to participate in an interactive exercise by submitting responses to questions around the prioritisation of intervention ideas via OMBEA, a web-enabled response option integrated with Microsoft PowerPoint. A total of *n* = 57 practitioners, mangers and leaders participated in the exercise.

In addition to the conference, we convened separate workshops at two timepoints during July and August 2023 with: (i) CYP in contact with child welfare services, and (ii) mental health and child welfare practitioners. The research team worked in partnership with the participants to iteratively co-produce the intervention. At timepoint 1, we presented the findings of WPs 1–3 and the prioritisation exercise to CYP (*n* = 6) and practitioners (*n* = 11). Workshop participants were encouraged to consider these findings during semi-structured activities, which were designed to support discussion relating to prioritised intervention ideas. Activities included listing strengths and weaknesses of the approaches, preferred mechanisms of change, and examining intended and unintended outcomes which may come from the mechanisms. At timepoint 2, a sub-group of participants attended a further workshop (*n* = 4 in CYP workshop; *n* = 4 practitioner workshop). These workshops focused upon refining the detail of the intervention and producing a detailed intervention logic model.

## Results

### Step 1: define and understand the problem

The first step of the 6SQUID approach is to clarify the ‘problem’. We commenced the formal intervention development process with a clear understanding that the mental health of CYP in contact with child welfare services is a priority public health and social care concern. This was informed by a review of existing evidence [4, 44–46] and stakeholder involvement within project design stage. What we did not yet know, and needed to clarify, was what the social distribution of this problem was within the population of CYP in contact with child welfare services. We were concerned with the future implementation of the intervention within the child welfare sector, and as such we sought to understand which groups of children within the larger population of CYP were of greatest concern to child welfare and mental health practitioners, and who they perceive to be most or least likely to benefit from intervention. We were also concerned to examine the causal pathways linked to mental health problems and determine which immediate (proximal) and underlying (distal) influence give rise to mental health problems in CYP in contact with child welfare services and in what ways these CYP are affected by mental health problems.

#### Childhood adversity

We drew upon the findings of the practitioner focus groups (WP2) and interviews with CYP and parents/caregivers (WP3) to examine the mental health needs of CYP in contact with child welfare services. Our application of the socio-ecological model highlighted the prevalence of interpersonal risk factors for CYP in contact with child welfare services. Adversity and trauma within the home environment was thought to be a particularly prominent risk to CYP’s mental health and a substantial priority issue for child welfare workers, CYP and parents/caregivers, and further reinforced by stakeholders. This distal, underlying influence of adversity typically consisted of parental risk factors such as parental mental health problems, parental substance use, domestic violence, and having a parent in prison. Participants highlighted the complexity within the context of adversity. Interpersonal risk factors were thought to interact synergistically with other risks present, resulting in accumulative stress for CYP in contact with child welfare services. Participants also reported a series of immediate, proximal influences. CYP were thought to often experience shame and stigmatisation relating to experiencing adversity and trauma within the home and would exhibit externalising difficulties including risk-taking or challenging behaviours as well as internalising difficulties such as low mood and self-esteem. These factors were reported to compound CYP vulnerability through exacerbating conflict in CYP-parent/caregiver interactions and increasing behavioural problems exhibited within other settings such as school and within local communities. Poverty was also highlighted as contributing to very difficult contexts for CYP and contributing to the impact of mental health problems within families.

*“It comes from home*,* as a result of really struggling to manage and move on*,* and recover from traumatic situations*,* and/or believing that they were the cause of those*,* in some way*,* shape or form. So*,* believing that they are bad*,* that they are the cause of difficulties within their family…if there’s a pervasive sense of shame that exists within the child*,* wherever that comes from*,* that essentially leads fundamentally to poor self-esteem*,* difficulties in relationships*,* and poor mental health outcomes” (female*,* child and adolescent mental health practitioner).*

*“Back at school when I got angry*,* I was taking it out on the wrong people*,* I was attacking my fellow pupils and that. Thinking about it now*,* I feel sorry that I was doing that*,* that I was using this anger and I was just using it against other people and hurting them*,* I was hurting my own teachers*,* I was attacking them and they had to restrain us. I feel guilty and I feel so sorry” (male*,* age range 11–13 years*,* child in care).*

#### Failing to recognise trauma

It was reported by participants that parents/caregivers and the professionals involved in their care often did not know how to best support the CYP or deal with their related behaviours. Participants highlighted that early ‘warning signs’ were typically overlooked resulting in missed opportunity to intervene before CYP developed diagnosable mental health disorders. Our findings further suggested that there is a tendency for early indicators to be perceived as ‘bad behaviour’, rather than being symptomatic of CYP’s experience of adversity. When the CYP’s mental health needs were identified, participants reported that care was further delayed by lengthy waiting lists for mental health treatment, leading to increased mental health vulnerability in CYP and a worsening of their social situation.

*“From my own experience. Growing up*,* there was a lot of issues raised to social services*,* to school and things and they*,* sort of*,* waited until it was at crisis point to actually do any intervention whatsoever. Whereas if I think*,* you know*,* if they’d came in and actually just tried to do little things earlier*,* it might not have got to the point it did”* (*female*,* 20 years*,* care leaver*).

*“The waiting list is huge. You’re talking over a year*,* to be able to just have an initial appointment. We waited 14 months. Bearing in mind*,* [daughter’s name] was already in CAMHS*,* but for an autistic assessment*,* we waited 14 months. In fact*,* I think it was longer*,* because COVID hit…And there’s nothing you can do except get on with it*,* and just try and struggle along” (female*,* mother*,* child in need)*.

#### Retraumatising

Within focus groups and interviews, participants frequently highlighted that when CYP attended mental health services they were required to retell their story as a prerequisite to receiving treatment, and without first paying adequate attention to building a trusting relationship. This was found to be unhelpful, and at times distressing for CYP. This typically led to CYP not disclosing their concerns to mental health professionals, and their needs going unmet.

*“Some of them come in as if you’re going to trust them straight away… When they come in the house and they just sit down and they’ll be like*,* “Tell me how you’re feeling. How are you feeling today? How have you been feeling lately?” I don’t open up as easy as they may have thought… [they should] probably try and build a relationship*,* doing something like even if it’s just going to the beach…and then we can build a relationship from there” (female*,* age range 14–17 years*,* child in need)*.

*“So*,* you take these extremely traumatised children to an appointment*,* and obviously*,* they don’t trust the professionals*,* and if the kids don’t engage in one or two sessions*,* then they close the case for them*” (*female*,* residential children’s home*,* registered manager*).

### Step 2: clarify which causal or contextual factors are malleable and have the greatest scope for change

#### Intervening early

Having developed an understanding of the proximal and distal influences upon the mental health of CYP in contact with child welfare services, our next task was to identify which of these factors are malleable to change. Our systematic review of reviews (WP1) found a large evidence-base suggesting secondary prevention targeting CYP who had experienced adversity is effective at reducing mental health problems. Similarly, interventions with CYP with subclinical externalising problems were found to offer promise. Within the review, both risk and resilience factors were found to be highly malleable to change. We presented findings from WP1-3 to practitioners within the prioritisation exercise conducted within WP4, and asked stakeholders to respond to questions about causal or contextual factors which the intervention should target. Stakeholders overwhelmingly (83%) opted for an intervention with CYP who had experienced adversity, and at a point before symptoms of mental health problems are evident. This was further considered within the co-production workshops with CYP in contact with child welfare and practitioners (WP4). Workshop participants agreed that intervening early in the disease trajectory (and before symptoms were evident) was important. Further, we identified convincing evidence of an association between CYP adversity and mental health problems identified within our on-going engagement with research literature [46–50]. This combined evidence supports the provision of a selective intervention for CYP in contact with child welfare services who have experienced adversity, without first requiring the CYP to make potentially distressing disclosures within an assessment of mental health need.

**Fig. 2:**
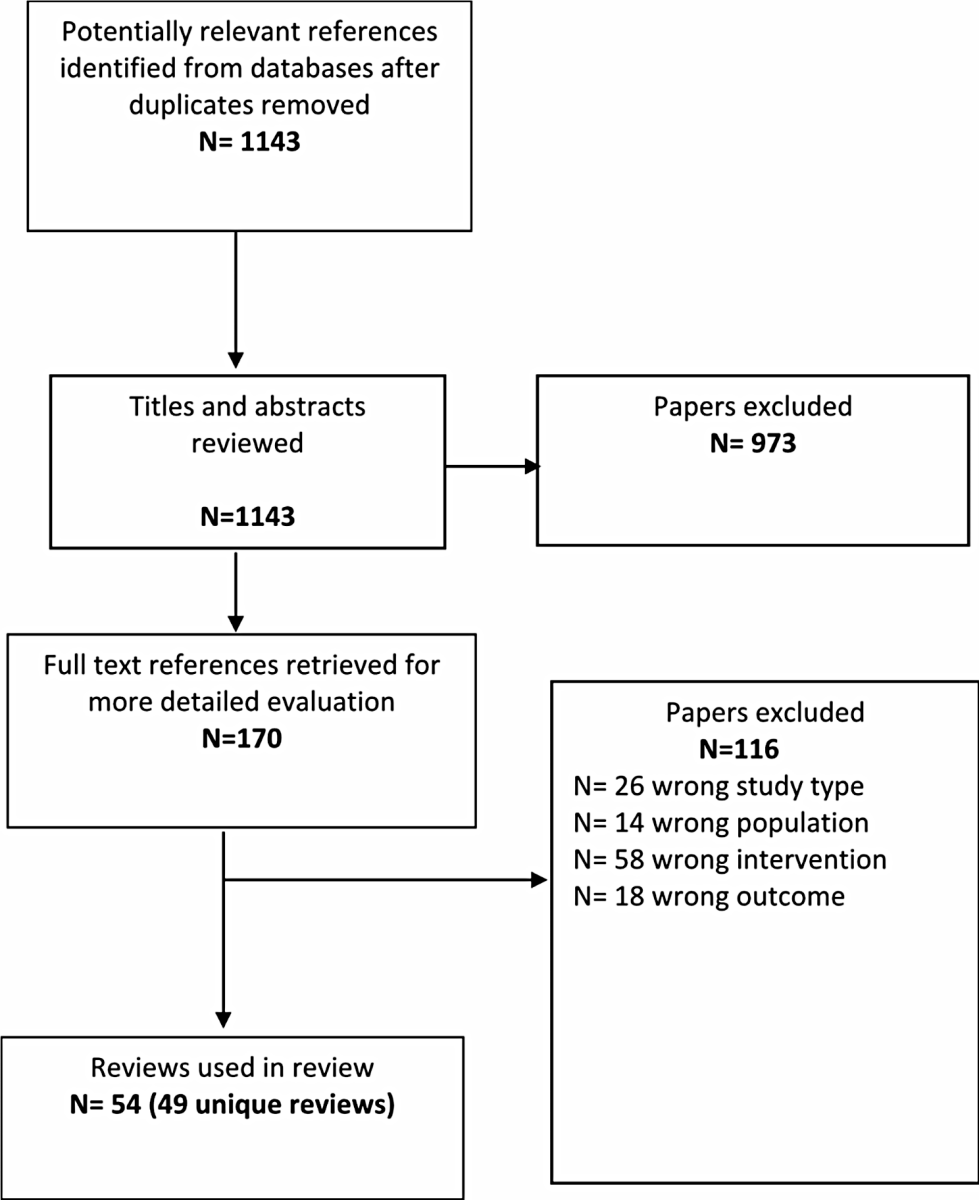
Flow of studies

#### Building supportive relationships

Throughout the qualitative work packages and in co-production workshops, participants focused upon the quality of the relationship and emotional support provided between the parent/caregiver and child. The home environment was perceived as an area where the scope for change was greatest both in terms of alleviating an important proximal factor, and the likely benefits of doing so. This was informed further by our consideration of research evidence showing an association between good CYP mental health and high emotional support, high parent-child closeness, and low parent-child conflict [51, 52]. Additionally, practitioners participating in focus groups within WP2 reported observing improvements in child mental health where parental support was high.

*“I think probably in terms of protective factors*,* I think probably the parents*,* in the sense of that’s where*,* you know*,* they’re in school all day*,* yes*,* but that’s where*,* like we’ve just said*,* from a young age as well*,* that’s where*,* kind of*,* they’re nurturing*,* where they’re learning*,* I think. Even if they were in school all day and had a great protective network*,* if they go home and there’s absolutely no protection there – for teenagers especially*,* no boundaries*,* no rules*,* no independent living skills*,* and all that*,* we’re on a losing battle if the parents aren’t*,* kind of*,* putting that in place 24/7”* (*female*,* care leavers team*,* social worker*).

The development of strong support systems such as supportive friendships and intimate relationships, wider family networks and positive relationships with professionals were also highlighted as important protective factors which could be fostered with the potential for substantial benefit for CYP.

*“It would be having a relationship with a positive role model adult in their life that allows them to see themselves in a bit of a different way. Because I guess a lot of the kids that we work with have had really difficult early relationships and difficult*,* maybe*,* parent/child relationships*,* or difficulties with other adults…So whether that’s a member of staff*,* or it could be a member of educational staff*,* it’s having that positive role model that maybe they haven’t had earlier on*,* so that they can start to develop those trusted relationships*,* be able to see themselves in a bit of a different way and start to internalise some of that”* (*female*,* child and adolescent mental health practitioner*).

### Step 3: identify how to bring about change: the change mechanism

Interventions are ‘theories incarnate’ [53] and may include implicit or explicit theory [33, 54]. It is recommended that intervention development draws upon established theory [30] which can support the identification of what is important, relevant and feasible in achieving the intervention goals [55]. During this project we were influenced by attachment theory and related attachment-focused interventions. Attachment theory explains how childhood adversity and trauma can reduce the security of attachments a CYP has with their caregiver [56]. In the early years, a child’s sense of safety and security comes primarily from their caregiver and they learn to trust/mistrust according to this experience [57]. Children who experience adversity and trauma experience the world as unsafe [58]. They learn that their caregiver cannot or does not protect them from this danger and they learn to mistrust [59]. In the absence of trusting and secure attachments, children’s development maybe organised around a nervous system which is prepared for danger [58]. This may result in emotional dysregulation [60] and behaviours which are deemed socially unacceptable [61]. This in turn compounds the problems of the CYP (for example generating conflict in the home of disruptive behaviours within school) [58], whilst also creating a barrier for help-seeking [62].

During stakeholder consultation and within co-production workshops we examined mechanisms of change. Attachment-focused interventions such as the Solihull Parenting Approach [63] and Dyadic Developmental Psychotherapy (DPP) [64] where highlighted as important mid-range theories [55] with explanatory potential relating to the change mechanism. Both approaches emphasise the importance of CYP feeling safe and developing relationships with key caregivers, before learning how to regulate emotion within the safety of those relationships. An additional mechanism of change includes recognising and understanding the impact of adversity and trauma upon the CYP’s feelings and behaviours. We were further influenced by the Solihull Parenting Approach which recognises parental anxiety as an important factor, and provides a means of the parent and child developing reciprocity [65] and reflexive functioning [66] in order to achieve change [63].

### Step 4: identify how to deliver the change mechanism

**Fig. 3:**
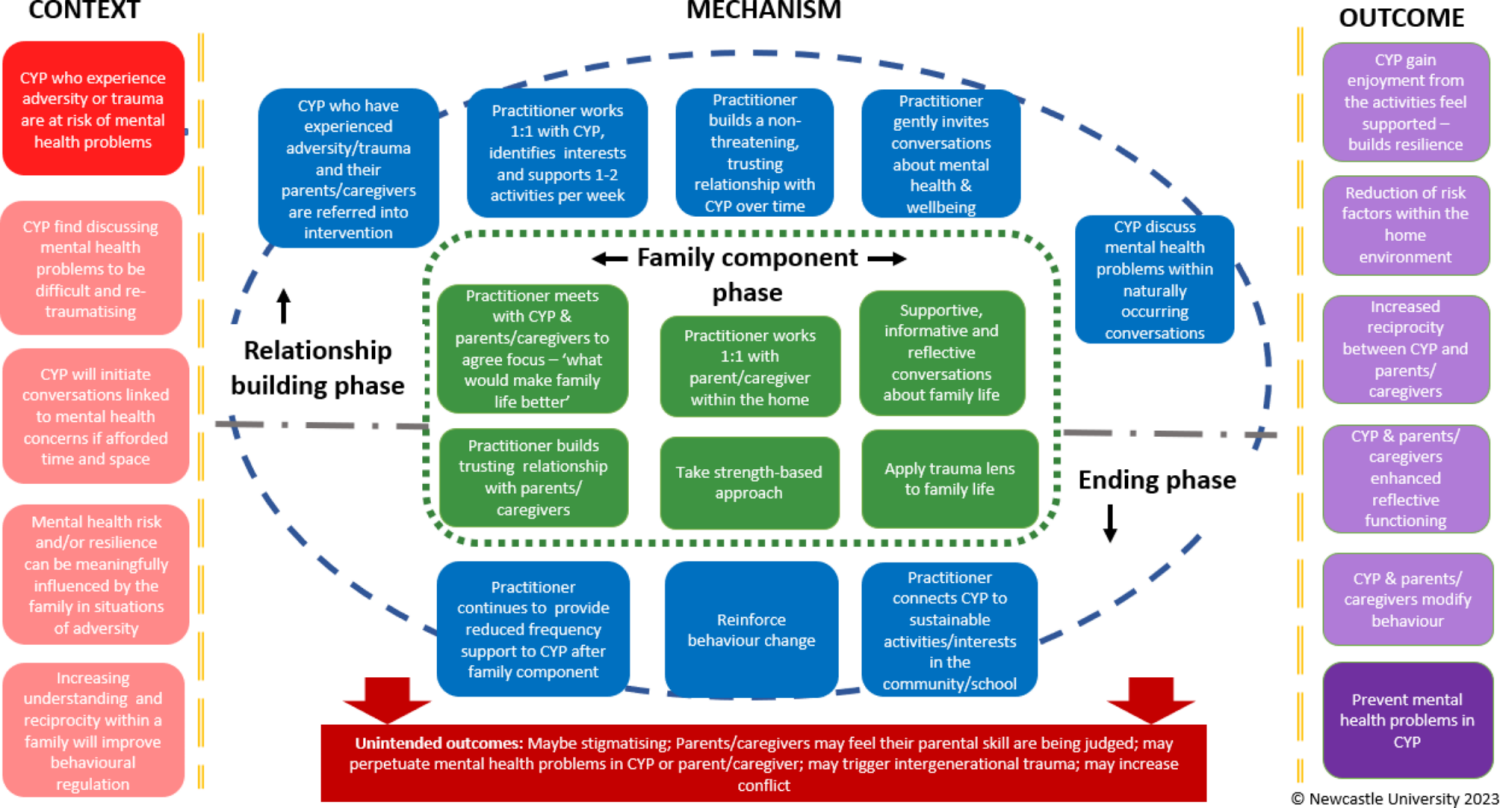
Intervention logic model

We iteratively developed and refined the intervention within a series of four co-production workshops (WP4). A detailed intervention logic model is presented in Fig. 3 wherein we depict how the intervention may work and its anticipated intended and unintended outcomes. Co-production workshops were based upon the outcomes of the prioritisation exercise and informed by the findings of work packages 1–3 and the iterative refinement of the previously described intervention development steps. Within the prioritisation exercise (WP4), practitioners were asked to rank their preferred three intervention approaches. Activity-based interventions with CYP, family/caregiver interventions and trauma-informed approaches were selected as the three highest priority approaches from a shortlist of 10 possible interventions.

It was agreed within co-production workshops conducted at timepoint 1 that any intervention with CYP who have experienced adversity should take a trauma-informed approach. Whilst there is no consensus on how trauma-informed care is defined [67], the most widely used definition of trauma-informed care comes from the Substance Abuse and Mental Health Services Administration who define the “Four R’s” of trauma-informed care, which are: realisation about trauma and its affects; recognition of the signs of trauma; responding to trauma and resist re-traumatisation through practices which inadvertently cause further trauma [68]. We proceeded to consider the strengths and weaknesses of the two remaining intervention approaches of activity-based intervention and family/caregiver intervention, with a view to agree which of these approaches was perceived to be the better approach. We moved back and forth between step 3 and step 4, refining our change mechanism and our approach to delivering this. During this iterative process, it became apparent that both approaches were considered necessary to bring about change within the mechanism and prevent mental health problems in CYP in contact with child welfare services. This led to a decision within the co-production workshops to develop a trauma-informed activity-based youth intervention with an embedded family component. The key characteristics of the intervention are:

- Selective secondary preventative intervention for CYP in contact with child welfare services who have experienced adversity (e.g. parental substance use, mental health, domestic violence, incarceration of a parent).
- A youth activity-based intervention which would consist of three phases:
- A relationship building phase (approx. 8–12 weeks): practitioner getting to know the CYP and their interests, agreeing and supporting involvement in a range of activities (e.g. going for walks, visiting places of interest, jointly researching topics of interest, attending organised activities).
- A family component phase (approx. 6–8 weeks) – see below.
- Ending phase (approx. 4–6 weeks): practitioner reinforces CYP learning and related behaviour change and supports access to youth activities and established networks in community.
- An embedded family component:
- Practitioner meets with CYP and family together and agrees plan for work.
- Practitioner has weekly sessions separately with CYP and parents/caregivers focused on supporting family members to understand each other; the emotional and behavioural impact of adversity on CYP/wider family and reflective family functioning.

## Discussion

This project has co-produced an intervention with, and been informed by, CYP in contact with child welfare services, their parents/caregivers and the practitioners who support them. In doing so, we have provided a unique contribution to an under-researched topic [11], of international importance [69, 70]. We identified that the primary risk factor affecting CYP in contact with child welfare services is the experience of adversity. The quality of relationships that the CYP experiences both with their parent/caregivers and the professionals involved in their care were considered the main factors amenable to change. By focusing upon building secure and trusting relationships with the CYP and promoting understanding how adversity and trauma impacts upon the CYP within the family, our project suggests the CYP mental health maybe best supported. The findings of our study have resulted in the development of a trauma-informed, activity-based intervention with an embedded family/caregiver intervention.

The importance of taking a trauma-informed approach to intervening with CYP in contact with child welfare was evident throughout our project. This finding is in line with emerging evidence supporting such practices with vulnerable populations of CYPs [71–75]. The CYP involved in our project emphasised the potential for existing mental health support to re-traumatise, as CYP are often required to repeatedly discuss distressing matters with unknown professionals within clinical interactions. Our findings emphasise that a trusting relationship with a familiar and non-threatening practitioner is necessary to allow CYP the space and opportunity to choose to talk about their mental health concerns at a pace that is comfortable to them. This supports previous intervention research with CYP in contact with child welfare services [76].

Both youth and family focused components were considered a priority within our co-production activities. As has been found in other studies of CYP experiencing adversity [77–79] and/or mental health problems [80], participants highlighted the need for CYP to be supported both separate to and within family structures. The initial phase of the intervention is an activity-based, youth focused intervention consisting of one-to-one interactions with a consistent practitioner with the primarily focus being upon building a relationship. This gradual building of rapport would enable the practitioner to facilitate naturally occurring conversations about mental health and concerns. After establishing a relationship with the CYP, this practitioner is then able to progress onto the family-focused component of the intervention; providing the consistent relationship that research highlights as particularly necessary with vulnerable groups of CYP [14, 81]. The family-focused component aims to improve the parent/caregiver-child relationship through increasing the reflective function (the capacity of family members to understand one another’s behaviour in-light of underlying mental states and intentions) and reciprocity; attributes that research has found to be beneficial to CYP mental health and wellbeing [66, 82]. However, a theme throughout our project was the importance of avoiding stigmatising practices and approaches which made parents/caregivers feel that their parenting skills were being questioned. As such, particular care is needed to be given to the relationship between the CYP, parent/caregiver and practitioner during an intervention in this context [76, 83].

### Strengths and limitations

To our knowledge this is the first project to co-produce a preventative intervention for CYP in contact with child welfare services with young people themselves, their parent/caregivers and the practitioners who support them. The meaningful stakeholder involvement we have achieved throughout the project is a great strength. Whilst we envisage that this approach has resulted in an intervention which is most likely to respond to the needs of CYP in contact with child welfare services, further research is required to pilot the intervention, refine it and determine effectiveness [31]. A further limitation of our study is that the sample was recruited exclusively from the North-East of England. Further research may be required to examine transferability to other areas of the UK.

## Conclusion

A trauma-informed, selective secondary preventative intervention consisting of an activity-based intervention with an embedded family-focused component maybe most likely to respond to the mental health needs of CYP in contact with child welfare services who have experienced adversity. Further research is needed to pilot this intervention and gather evidence of its effectiveness.

### Electronic supplementary material

Below is the link to the electronic supplementary material.

Supplementary Material 1

## Data Availability

The research team do not have ethical approval to share data.
