# Supplementary material for: Co-producing an intervention to prevent mental health problems in children and young people in contact with child welfare services
Source: BMC Public Health. 2024 Aug 21;24:2276. doi: 10.1186/s12889-024-19770-6 (PMC11340120; doi:10.1186/s12889-024-19770-6)

Practitioner focus group topic guide (WP2)

- What are the mental health needs of children in contact with social care services?
  - Probe around concerns below diagnostic threshold, different populations of children (e.g. ages, reasons for contact with social care)
- How do you think mental health problems impact the children you support?
  - Probe around concerns below diagnostic threshold, different populations of children (e.g. ages, reasons for contact with social care)
- What do you think are the main contributory factors to mental health problems amongst these children?
  - Probe around concerns below diagnostic threshold, different populations of children (e.g. ages, reasons for contact with social care)
- How are these children currently supported by social care/early help services?
- We'd like to think about some particular groups of children you might support in your work. What are their particular mental health concerns, mental health needs and mental health risk factors for children in contact with social care that concerns you most in your work?
  - Probe around :
  - Children receiving care due to adolescent factors – i.e. behavioural problems; educational and learning difficulties, disabilities, self-harm
  - Children referred due to parental factors – i.e. domestic violence, parental mental health, substance use
  - Children who had experienced abuse/neglect
- Of the factors you've identified that are risk/protective factors for mental health problems amongst the children you support, which do you think are modifiable/could be changed?
  - Probe specifically modifiable to change through non-specialist intervention i.e. via support provided within social care, probe which could legitimately and adequately addressed with some support within social care/non-specialist setting and what could not
- Of these factors, which do you think are most important?
  - Probe whether they perceive children and families having similar or different view on what is important to address
- How might early identification/mental health support be strengthened within social care/early help service? (for children who do not meet the threshold for specialist support)
  - what could address the changeable risk/protective factors you identified?
  - How do you think these approaches might make a difference to these children/young people?
  - Which of these approaches do you think could feasibly be implemented within services to prevent mental health problems amongst these children?

Parent/caregiver interview topic guide (WP3)

- Can you tell me about your experiences with Early Help/children's social work services (probe reason for involvement)?
- How is your family life (probe family composition and child(ren)'s emotional including behavioural, social, physical wellbeing)?

- How does your child(ren)'s mental health and wellbeing affect them? (probe around what is of greatest concern/impact for the child)
- How does your child(ren's) mental health and wellbeing affect the family? (probe around what is of greatest concern/impact for the family)
- What is your experience of current service provision in responding to your child(ren)'s mental health and wellbeing needs (probe around strengths and weakness of provision)
- What do you see as the main factors that affect your child(ren)'s mental health and wellbeing? (probe around the factors that are malleable to change)
- What might help your child(ren) with their mental health and wellbeing (probe around specific types of help, in what way they might help and who might be able to provide that help)
    - *Disclose some ideas practitioners came up with*
    - *Share also some findings from literature review*

CYP topic guides (WP3)

VIGNETTES EXERCISE

I'd like to talk a bit about other children who get help from a social care/early help worker. I have some made up stories about children who get help from a social care worker (show visuals and read the vignette to the young person).

1. What do you think is happening for this young person? (Prompt around school, family, friends, service involvement, other)
2. What things in this young person's situation do you think might make them feel angry, worried or sad? (Prompt around the reasons for this)
3. What things in this young person's current situation do you think might make them feel happy? What might help the young person with the anger worry or sadness they feel?
4. What new things do you think could be done that might help them to feel better when they feel like this?
5. Who might be able to help? (At what point)
6. In what way could they help? (how much would be needed to intervene)

HEADS EXERCISE

I'd like to talk about some ways you might have felt before. We have a selection of different heads to show different things you might have felt (angry, worried, sad…). Have you ever felt any of these feelings? Which one of these feelings have you feel most often?

- On the sheet there are a list of different things that sometimes make young people feel this way. Are there any of those words that describe things that have made you feel angry, worried or sad?
    - What is it about those things that have made you feel that way?
    - What happens when you feel angry, worried or sad? (prompt around how it affects the young person, others around them and specific aspects of their life e.g. school, friendships etc

- On the sheet there are a list of different things that some young people find have helped them at times when they have felt angry, worried or sad. Do any of those words describe things that have been helpful to you?
    - In what way did they help?
    - In what way could they have been more helpful to you
- What new things might have been done that would have been helpful to you when you felt angry, worried or sad?
- In what way do you think your social worker/early help worker (use practitioner's name) could have helped you at that time, or since that time to stop you feeling angry, worried or sad?

Case vignettes (used with CYP interviews; WP3)

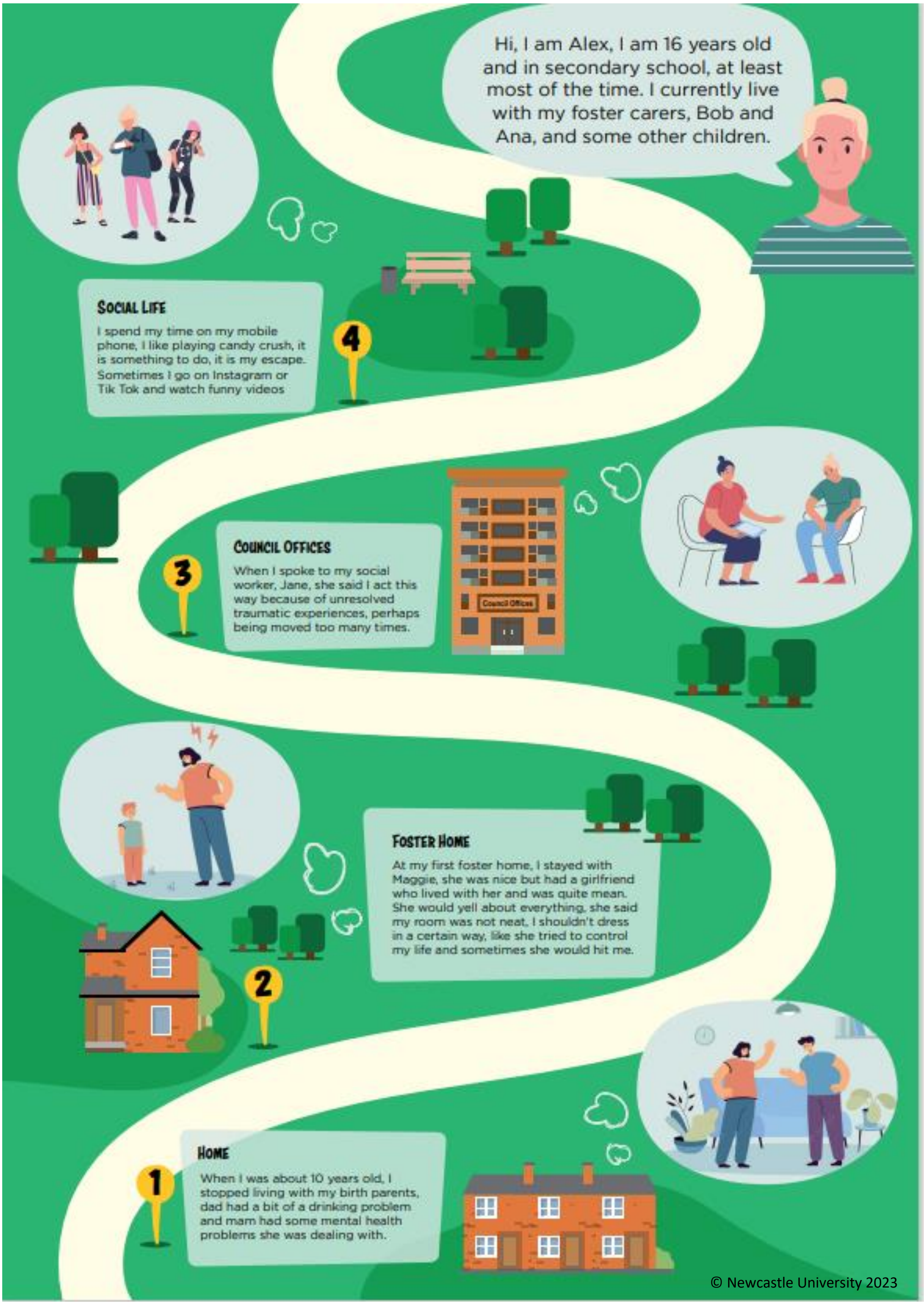

© Newcastle University 2023

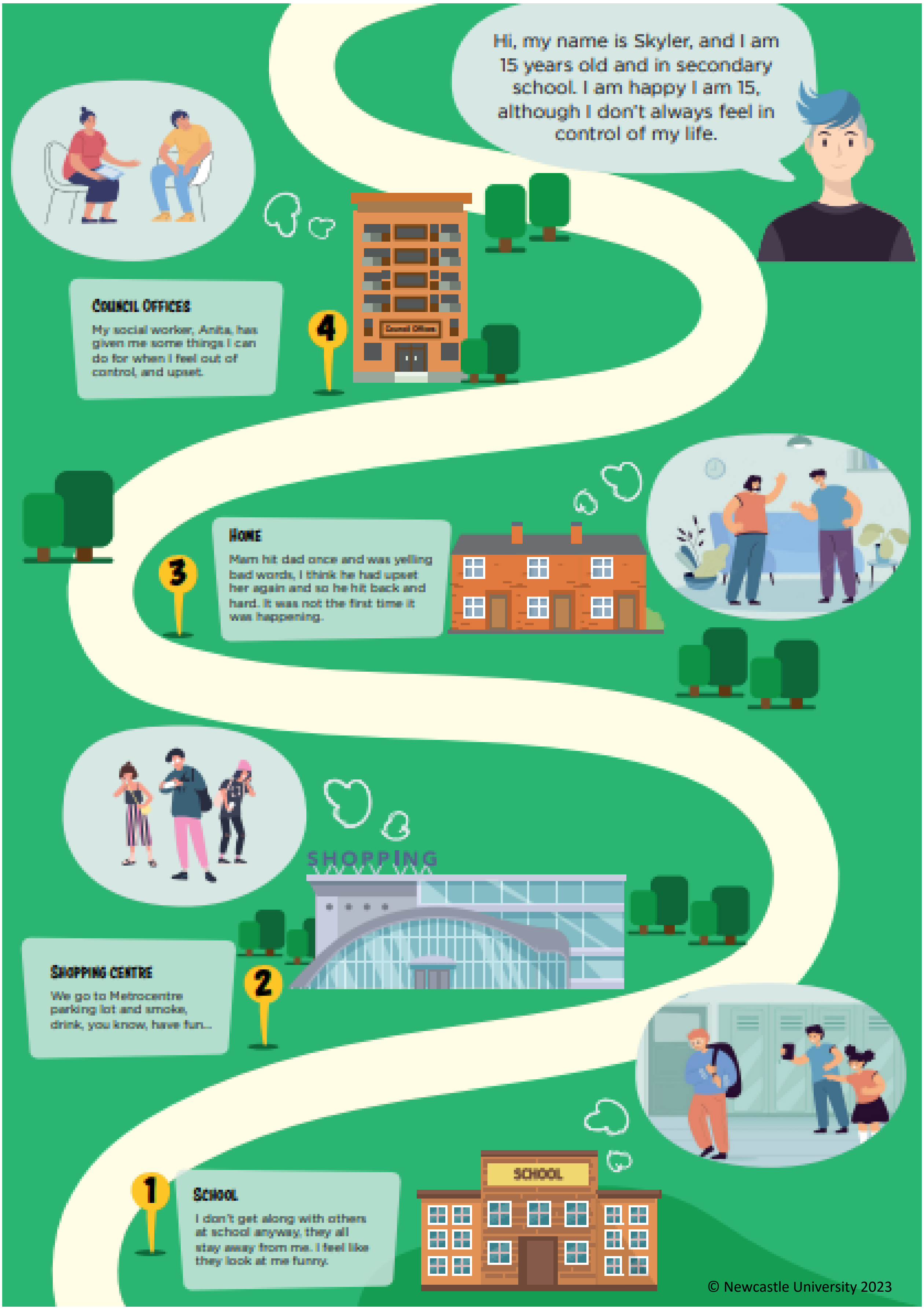

© Newcastle University 2023

Charlie aged 15

Hi, I'm Charlie, I'm 15 years old. I live with my mam, my dad used to be here but him and mam split up.

1

Home

Sometimes I miss him, but I don't miss the arguing, and the way mam would always be hurt afterwards and dad would be sad and sorry. He did other bad things as well and in the end Dad went to prison. That was a tough time.

2

Nana's

I used to go to Nana's sometimes but since she died I haven't gone many places. That was the worst thing that's happened, she was my shining star.

3

SCHOOL

School

I'm meant to be in school but I don't go much, I just hate it. It makes me sick to my stomach thinking about going into school, into lessons, trying to be friends with people I don't know and who probably don't like me. I feel like there's loads of things I can't do, sometimes I can't stop feeling sad, can't stop feeling worried about things, can't keep my temper under control.

4

Council Offices

Council Offices

My Social Worker says maybe social media is bad for me sometimes, she's probably right. I don't always eat a lot and seeing people's posts about themselves and their perfect bodies online sometimes makes me want to eat even less. **And I think, what should I do?**

© Newcastle University 2023

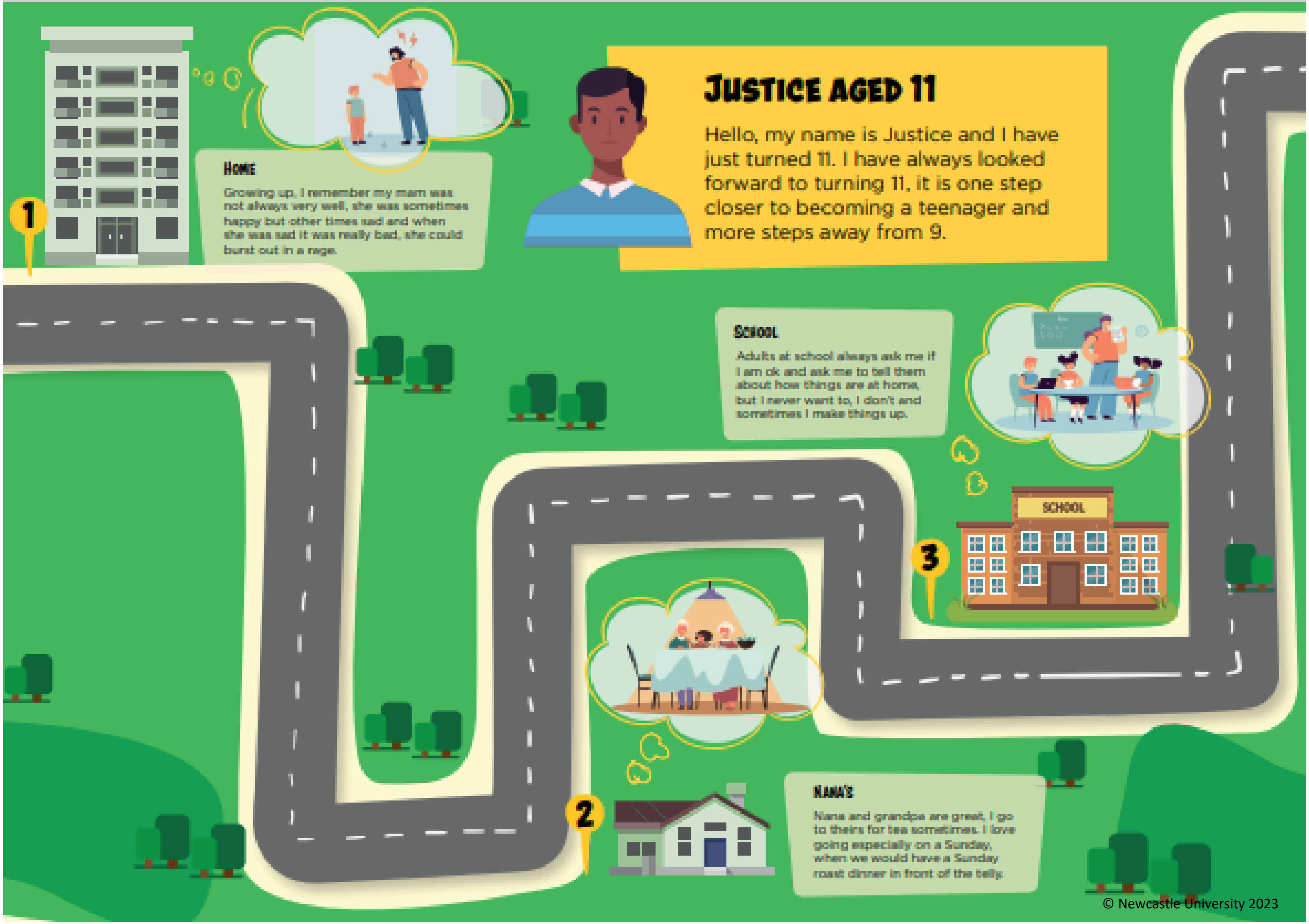

© Newcastle University 2023

Optional exercise (used in CYP interviews; WP3)

DOCUMENT 18 The WELLBEING Study; head exercise 'angry', Version 1.0. 22.11.21.
IRAS Project ID: 308660

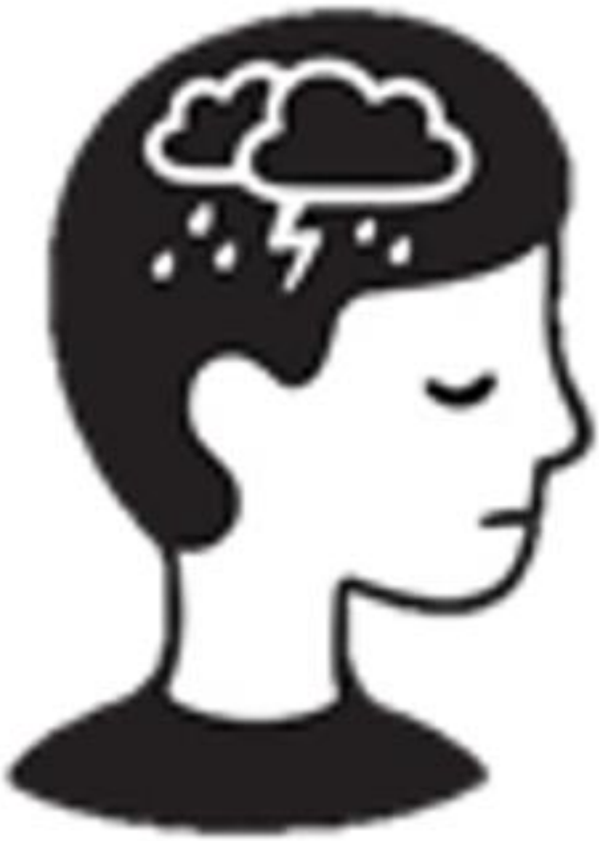

Things that might make someone feel this way:

Home family School/college friends thinking about past

Thinking about the future Anything else?

Things that did/may have helped when you felt that way:

Home family school/college friends activities having someone to talk to support from a worker anything else?

Supplementary figure: Thematic map (WP 2 and 3)

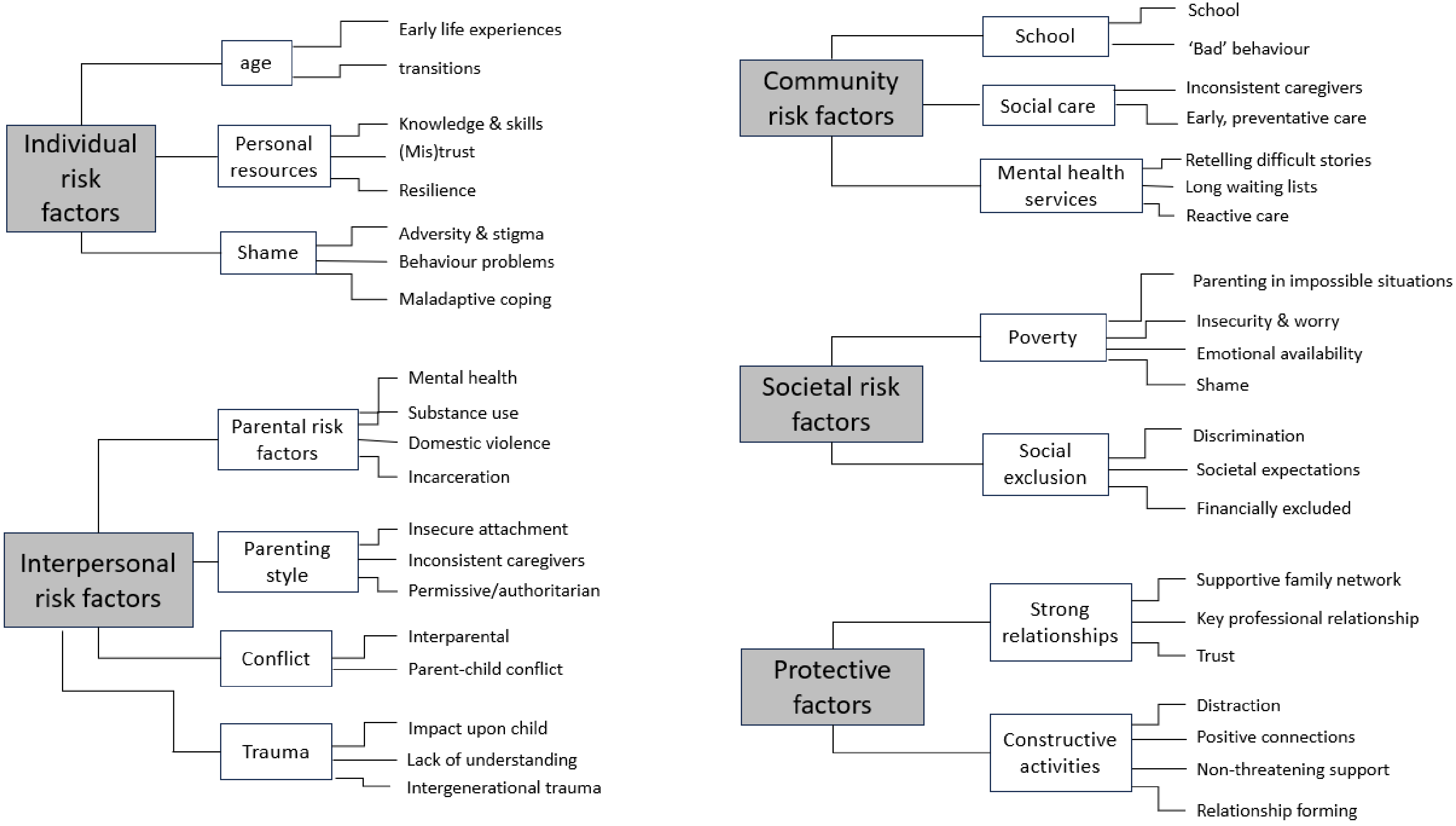

Supplement: Supplementary file 1 — Supplementary Material 1 [file 12889_2024_19770_MOESM1_ESM.pdf]
